# Analysis of microRNA regulating cell cycle-related tumor suppressor genes in endometrial cancer patients

**DOI:** 10.1007/s13577-020-00451-6

**Published:** 2020-10-29

**Authors:** Łukasz Witek, Tomasz Janikowski, Iwona Gabriel, Piotr Bodzek, Anita Olejek

**Affiliations:** 1 Department of Gynecology, Obstetrics and Oncological Gynecology, Medical University of Silesia, Bytom, Poland; 2 Silesian College of Medicine, Katowice, Poland

**Keywords:** Cancer, Micro RNA, Endometrial cancer, Tumor suppressor genes, Cell cycle

## Abstract

Endometrial cancer remains the most common malignancy of the female genital system in developed countries. Tumor suppressor genes are responsible for controlling the cells fate in the cell cycle and preventing cancerogenesis. Gene expression affects cancer progression and is modulated by microRNAs defined as both tumor suppressors and oncogenes. These molecules indirectly regulate multiple processes like cell proliferation, differentiation and apoptosis. The aim of this study was to analyze miRNAs expression that can regulate the activity of tumor suppressor genes related to the cell cycle in patients with endometrioid endometrial cancer. The study group consisted of 12 samples that met the inclusion criteria from a total of 48 obtained. The 12 samples were used to analyze microRNA expression. Complementary miRNAs were identified using TargetScan Database and statistical analysis. MicroRNAs were determined for the tumor suppressor genes: CYR61, WT1, TSPYL5, HNRNPA0, BCL2L1 and BAK1. All the miRNAs were complementary to the described target genes based on TargetScan Database. There were five miRNAs differentially expressed that can regulate tumor suppressor genes related to the cell cycle. The distinguished miRNAs: mir-340-3p, mir-1236-5p, mir-874-3p, mir-873-5p.2 and mir-548-5p were differentially expressed in endometrial cancer in comparison to the control. Among the distinguished miRNAs, the most promising is mir-874-3p, which may have an important role in endometrial adenocarcinoma proliferation.

## Introduction

Endometrial cancer (EC) remains the most common gynecological malignancy in developed countries, with more than 6000 newly diagnosed cases in Poland per year and 288 000 worldwide [1, 2]. The two main histological types of this cancer, endometrioid and non-endometrioid, present unique molecular aberrations and are responsible for disparate clinical behaviors [3]. Type I endometrioid endometrial cancer (EEC), including G1 and G2 adenocarcinoma, arises from atypical endometrial hyperplasia and are pathogenetically related to unopposed estrogen stimulation. This type of EC occurs in peri- and postmenopausal age, with abnormal uterine bleeding in 90% of the patients. As endometrioid cancer is diagnosed at early stages, it presents a relatively good prognosis. Type II includes grade 3 endometrioid tumors as well as non-endometrioid morphology (NEEC) and carries a poor prognosis. It is characterized by non-estrogen dependency and develops from atrophic endometrium [4].

MicroRNAs (miRNAs) are small, 19–25 nucleotide long, non-coding RNA molecules, which have emerged as fundamental, posttranscriptional regulators of protein expression by binding to a complementary sequence in the target gene. In cancer cells some miRNAs function as oncogenes, others as tumor suppressors [5, 6]. Tumor suppressor genes (TSGs) play a critical role in controlling the cell cycle assuring proper proliferation and differentiation. Thus, these genes prevent the accumulation of mutations and protect the cell from acquiring cancer phenotype by inducing apoptosis [7]. Progression in the cell cycle is regulated by cyclins and cyclin-dependent kinases (CDK). This process is controlled at three checkpoints, which the cell may pass to the next phase or can be halted [8]. Many internal and external factors, including TSGs and miRNAs can affect the cell cycle by inhibiting or initiating cell division [9]. Incorrect functioning of the cell cycle can affect multiple processes and has an impact on patient treatment mostly by drug resistance [10]. In a previous work, we examined the gene expression of tumor suppressor genes related to the cell cycle [11].

The aim of this study was to analyze miRNAs expression that can regulate the activity of tumor suppressor genes related to the cell cycle in patients with endometrioid endometrial cancer.

## Material and methods

### Patients

The study group consisted of 12 endometrial specimens with pathologically confirmed adenocarcinoma endometrioides. A total of 48 endometrial samples were obtained from patients surgically treated at the Department of Gynecology, Obstetrics and Oncologic Gynecology, at the Medical University of Silesia in Katowice, Poland between years 2016 and 2018, but most of them did not meet the inclusion criteria. The study was approved by the Institutional Review Board of Medical University of Silesia (KNW/022/KB1/64/10). All women underwent abdominal or vaginal hysterectomy. Clinically the cancer was classified according to the FIGO criteria. All patients had primary endometrial cancer and did not receive chemotherapy or radiation therapy prior to surgery. The reference group was comprised of endometrial samples obtained from women with diagnosed uterine fibroids, benign ovarian tumors or prolapsed uterus with morphologically confirmed endometrium in the proliferative phase. Patients with hormone therapy for the past 12 months, severe obesity (BMI > 30), endometriosis or adenomyosis, non-endometrioid endometrial cancer, adenocarcinoma with squamous elements, coexisting cervical cancer were excluded. The clinical characteristics of patients enrolled in the molecular analysis are presented in Table 1.

**Table 1:** Patient characteristics

Grade	Numer of patients	Avg. age ± standard deviation	Avg. weight ± standard deviation	BMI	Numer of pregnancies
Control	4	57.2 ± 9.11	80.3 ± 20.1	28.91	2.2
G1	3	67 ± 6.1	82.7 ± 17.5	29.88	2
G2	5	69.3 ± 7.6	83.9 ± 22.9	29.95	2.1

### Sample classification and storage

All analyzed tissue biopsies were collected after cutting the uterus in its sagittal plane, following the removal of the uterus via laparotomy or vagina. The obtained tissue samples (approximately 1 cm) were divided into two parts and placed separately in buffered formalin for pathomorphological confirmation and RNAlater (Life Technologies) solution for molecular analysis according to the producer’s instructions. Histological examination was performed according to WHO guidance.

### Total RNA isolation

The samples, which were obtained surgically, were homogenized. Afterwards, total RNA was extracted from endometrial specimens using TRIzol reagent (Invitrogen, Carlsbad, CA) according to the manufacturer’s instructions. RNA extracts were treated with DNase I to eliminate DNA (RNaesy Mini Kit, Qiagen, Valencia, CA). Isolated RNA was checked with the use of a spectrophotometer GeneQuant II RNA/DNA calculator (Pharmacia Biotech, Cambridge, UK). Next quality analysis was performed using 1% agarose electrophoresis stained with ethidium bromide. Only the positive outcome of both analyses was considered to be a qualifying result for further investigation via MiRNA 2.0 microarray (Affymetrix Inc., California, USA).

### MiRNA 2.0 microarrays

The first step for the miRNA 2.0 analysis was the poliadenylation of LMW (low molecular weight) with FlashTag Biotin HSR RNA Labeling Kit (Affymetrix, Ca, USA) of 750 ng from whole-cell RNA. The next step was ligation with the use of FlashTag Biotin HSR Ligation Mix and 2 µl ligase DNA T4 afterwards adding 2.5 µl HSR Stop Solution. At this phase 2 µl have been taken to perform ELISA QC Assay (FlashTag Biotin HSR RNA Labeling Kit; Affymetrix, Ca, USA). ELISA has been performed according to the producers manual and scanned with WallacWallac 1420 VICTOR (PerkinElmer). Hybridization of the mi RNA 2.0 array was done according to the producers manual (Affymetrix, Ca, USA). Staining was done using Fluidics Station 450 (Affymetrix, Ca, USA) and Hybridization Wash and Stain Kit (Affymetrix, Ca, USA). Scanning of the microarray was performed with the use of GeneChip Scanner 3000 7G (Affymetrix, Ca, USA) and Affymetrix^®^ GeneChip^®^ Command Console^®^ Software (AGCC).

### Statistical analysis

The statistical analysis was performed with ANOVA and post hoc Tukey HSD in Transcriptom analysis Console (Affymetrix CA. US) software. Databases such as Targetscan, Netaffx were used to determine the correlation between mRNA genes and miRNAs.

## Results

**Fig. 1:**
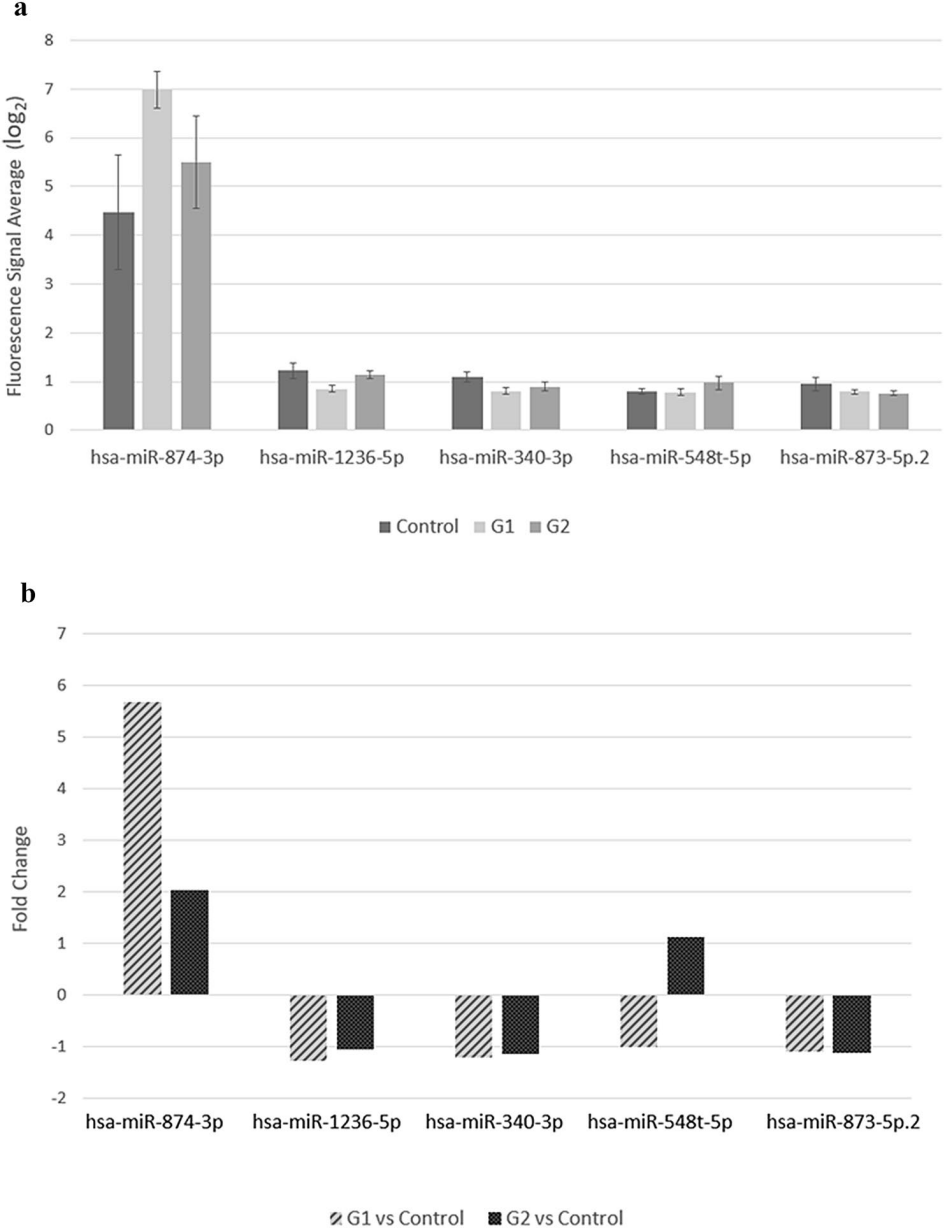
Expression of selected miRNA from ANOVA with post hoc Tukey HSD **a** average fluorescence signal with STD bars, **b** fold change values for differentially expressed microRNA

The statistical analysis of microRNA was performed using ANOVA and post hoc Tukey HSD. 1295 miRNA transcripts were obtained as statistically significant for endometrial cancer in comparison to the control. Expression of differentiated tumor suppressor genes regulating the cell cycle CYR61 (Cysteine-rich angiogenic inducer 61), WT-1 (Wilms Tumor 1), TSPYL5 (Testis-Specific Y-Encoded-Like Protein 5), HNRNPA0 (Heterogeneous Nuclear Ribonucleoprotein A0), BCL2L2 (BCL2-Like 2) and BAK1 (BCL2-Antagonist/Killer 1) was performed in a previous study. The presented mRNA genes were used to find microRNAs in Target Scan database that can potentially regulate their expression. Afterwards statistically significant miRNAs were selected using ANOVA with post hoc Tukey HSD (Table 2). Among the distinguished mRNA genes the highest number of miRNAs was found for HNRNPA0 counting 2579 Id miRNA, but only 41 were statically significant in the obtained results. The lowest number of statistically significant miRNA was identified for TSPYL5, only 23 differentially expressed microRNAs. The next step was to assess which statistically significant miRNAs were able to interact with all distinguished tumor suppressor genes regulating the cell cycle. Only five micro RNAs (mir-340-3p, mir-1236-5p, mir-874-3p, mir-873-5p.2 and mir-548-5p) met the criteria (Table 3). Fold change was calculated for each endometrial cancer grade (Fig. 1) with the highest value for miR-874-3p, which was overexpressed in G1 and G2 cancer with an FC of 5.69 and 2.09 respectively. Based on TargetScan Database results, these miRNAs were characterized by seed match and context score in percentile. Predicting the probability in which the target genes can be regulated (Table 4). According to Target Scan database, miR-1236-5p had the highest prediction score for BAK1, BCL2L2 and HNRNPA0. Based on the results mir-1236-5p had the highest probability of matching all target gene transcripts. For CYR61 the high prediction had miR-874-3p, furthermore, this micro RNA had a good prediction value for all miRNAs except WT-1. However, for WT-1 and TSPYL5 none of the selected microRNA had a prediction value above 90, whereas TSPYL5 had an overall lowest.

**Table 2:** Number of miRNAs potentially regulating cell cycle tumor suppressor genes from ANOVA with post hoc Tukey HSD

Gene symbol	Targetscan total	Statistically significant	Endometrial cancer stage	
			G1	G2
CYR61	942	25	7	19
WT-1	2120	33	9	22
TSPYL5	1100	23	6	17
HNRNPA0	2579	41	10	32
BCL2L2	1644	35	9	26
BAK1	1520	30	13	22

**Table 3:** Expression results from ANOVA with post hoc Tukey HSD for selected miRNA

miRNA	Endometrial Cancer Grade					
	G1 vs control			G2 vs control		
	*P* value	FC	Reg	*P* value	FC	Reg
hsa-miR-340-5p	0.014	− 1.22	↓	0.025	− 1.15	↓
hsa-miR-1236-5p	0.016	− 1.29	↓	0.412	− 1.05	↓
hsa-miR-874-3p	0.016	5.69	↑	0.469	2.04	↑
hsa-miR-873-5p.2	0.147	− 1.11	↓	0.038	− 1.13	↓
hsa-miR-548t-5p	0.926	− 1.01	↓	0.049	1.13	↑

**Table 4:** Characterization of selected miRNA based on target scan

Gene	hsa-miR-340		hsa-miR-1236		hsa-miR-874		hsa-miR-873		hsa-miR-548t	
CYR61	7mer-1A	51	7mer-1A	32	7mer-m8	95	7mer-1A	56	8mer	86
WT-1	7mer-m8	76	7mer-m8	86	7mer-m8	33	7mer-m8	48	7mer-1A	52
TSPYL5	7mer	51	7mer-m8	70	7mer	75	7mer-1A	76	7mer-m8	36
HNRNPA0	7mer-1A	51	8mer	94	7mer-1A	53	8mer	92	7mer-1A	52
BCL2L2	7mer-1A	51	7mer-m8	90	7mer-m8	88	7mer-m8	49	7mer-m8	64
BAK1	7mer-1A	51	8mer	96	7mer-1A	73	7mer-1A	51	7mer-1A	89

## Discussion

Endometrial cancer remains the most common malignancy of the female genital system in developed countries [12]. Gene expression patterns indicate the state of ongoing processes in the cell and shed new light on cancer pathogenesis. However, our understating of microRNA role in endometrial cancer is still limited. The miRNA mechanism of action establishes a complex cell environment just by the fact that one gene can be targeted by multiple molecules. This is becoming more complicated by molecular interactions between enzymes and signaling pathways influencing cell processes such as proliferation, apoptosis or the cell cycle. This lead to the necessity of implementing statistical and bioinformatical analysis to predict the possible impact of miRNAs on patient treatment and prognosis [13].

The study focuses on evaluating miRNA related to tumor suppressor gene expression in polish endometrial cancer patients. Similar studies outlining the expression of miRNA in endometrial cancer patients have been performed around the world giving various results [14]. Based on our pervious results, exploring TSG expression we identified miRNAs that can regulate CYR61, WT-1, TSPYL5, HNRNPA0, BCL2L2 and BAK1 [11]. The study was performed on 12 patient samples that met demanding including and excluding criteria. This enabled an accurate view of molecular changes occurring in endometrial cancer cells. The statistical analysis showed several miRNAs significant for G1 and G2 EEC, which can regulate target tumor suppressor genes. Grade 3 EEC was not included in the analysis because of failure to meet the criteria. The identified miRNAs (mir-340-3p, mir-1236-5p, mir-874-3p, mir-873-5p.2, mir-548-5p) were complementary to all target genes and may potentially regulate their expression. Among the differentially expressed miRNAs: miR-340-3p, miR-1236-5p and miR-873-5p.2 were down-regulated regardless of tumor grade. Furthermore, all of them had a similar fold change level. The hsa-mir-548-5p expression was diverse in each cancer grade. In G1 cancer was decreased, in contrast it was increased in G2 tumor samples. Over-expression was observed only for hsa-mir-874-3p in both cancer grades in comparison to the control.

Xie et al. in 2018 showed that miR-340-3p influences Bax pro caspase 3, p27 and BCL-2 protein expression. Over-expression of the microRNA led to decrease of Bax pro caspase 3, p27, in turn, expression of Bcl-2 was silenced in endometrial cancer cells inducing apoptosis. Inhibition of miR-340-3p had an opposite effect increasing Bax, pro caspase 3, p27 and decreasing Bcl-2 expression in consequence favoring survival and proliferation. The described observation indicates the significance of miR-340-3p in the cell cycle [15]. This is enforced by the fact that in breast cancer miR-340-3p had an impact on chemotherapy, where low expression of the miRNA had a negative predictive value for patients [16, 17]. In our study miR-340-3p was down-regulated in cancer tissues. The TargetScan Database showed the highest affinity between miR-340-3p and WT-1, whereas for other distinguished TSG it was only 51. This further confirms the anti-oncogenic role of miR-340-3p in cancer cells and suggests its importance in the cell cycle. Similar expression patterns were noted for miR-873-5p.2, which had the highest relative score value for HNRPN0. The mRNA may indirectly affect DNA repair. However, the role of miR-873-5p.2 was mostly correlated with embryogenesis and development pathways like Hedgehog [18]. Another silenced micro RNA was miR-1236-5p. In contrast, this miRNA presented overexpression in other cancer tissue. Up-regulation in hepatocellular carcinoma led to G1/S cell arrest by acting on the P13K/AKT pathway [19]. A similar effect was observed in bladder cancer, where mir-1236-5p stopped the cell cycle in S phase by influencing F-box protein S-phase kinase-associate protein 2 (Skp2) expression [20]. In contrast, miR-1236-5p was silenced in ECC and had an opposite effect on the cell cycle. This would correlate with mRNA expression. BCL2L2, BAK1, HNRNPA0, WT-1 and TSPYL5 are known for regulating the cells cycle in the G1/S checkpoint. Alteration in their gene expression can lead to apoptosis or increased proliferation.

The miR-548 family was the only micro RNA that presented opposite expression in different cancer grades. In G1 cancer gene expression was down-regulated, whereas in G2 it was up—regulated. Based on TargetScan miR-548-5p had the highest prediction ratio for CYR61 and BAK1 genes. Taking into consideration the mRNA expression results for these genes it is possible that miR-548-5p can effect BAK1 because both molecules presented the same expression pattern. However, in the case of CYR61 the effect can be opposite, where miR-538t-5p may silence its expression. There are limited information about miR-548-5p in literature, but members of this multi-copy microRNA family have low expression patterns in tumor tissue. Suggesting that miR-548-5p can be treated as an anti-oncogenic miRNA in lung cancer [21]. In turn, expression of miR-548-5p was high in estrogen-positive breast cancer [22]. It is unclear if the expression of this miRNA can be estrogen related and further studies are required.

Overexpression in both endometrial cancer grades was observed only for miR-874-3p. This micro RNA had a high prediction ratio for all distinguished tumor suppressor genes. However, TSG expression results suggest a high probability of a silencing effect on CYR61 and TSPYL5, because of their decreased expression in grades 1 and 2. In osteosarcoma miR-874-3p was down-regulated leading to cyclin E1 increased expression. It was observed that the elevation of miR-874-3p inhibits CCNE1 expression in osteosarcoma, which led to G1/S arrest [23]. Furthermore, in breast cancer, increased expression of miR-874-3p inhibited cell proliferation by suppressing cyclin-dependent kinase 9 (CDK9) protein level [24].

Decreased expression of miR-874-3p was noted in several cancer tissues (breast, gastric, head, neck), thus leading to the assumption that the micro RNA can act as an anti-oncogenic factor inhibiting cancer progression.

## Conclusion

There were five miRNAs differentially expressed that could potentially regulate tumor suppressor genes related to cell cycle expression. Among the distinguished miRNAs the most promising is miR-874-3p, which may have an important role in endometrial adenocarcinoma proliferation and cell cycle regulation.

## References

[1] Makker A, Goel MM (2016). Tumor progression, metastasis, and modulators of epithelial-mesenchymal transition in endometrioid endometrial carcinoma: an update. Endocr Relat Cancer.

[2] Wojciechowska, Urszula D (2017). Zachorowania i zgony na nowotwory złośliwe w Polsce.

[3] Bokhman Jv (1983). Two pathogenetic types of endometrial carcinoma. Gynecol Oncol.

[4] Amant F, Mirza MR, Koskas M, Creutzberg CL (2018). Cancer of the corpus uteri. Int J Gynaecol Obstet: The Off Organ Int Fed Gynaecol Obstet.

[5] Macfarlane L-A, Murphy PR (2010). MicroRNA: biogenesis, function and role in cancer. Curr Genomics.

[6] Liolios T, Kastora SL, Colombo G (2019). MicroRNAs in female malignancies. Cancer Inform.

[7] Wang L-H, Wu C-F, Rajasekaran N, Shin YK (2018). Loss of tumor suppressor gene function in human cancer: an overview. Cell Physiol Biochem: Int J Exp Cell Physiol Biochem Pharmacol.

[8] Zheng K, He Z, Kitazato K, Wang Y (2019). Selective autophagy regulates cell cycle in cancer therapy. Theranostics.

[9] Otto T, Sicinski P (2017). Cell cycle proteins as promising targets in cancer therapy. Nat Rev Cancer.

[10] Si W, Shen J, Zheng H, Fan W (2019). The role and mechanisms of action of microRNAs in cancer drug resistance. Clinical epigenetics.

[11] Witek L, Janikowski T, Bodzek P, Olejek A, Mazurek U (2016). Expression of tumor suppressor genes related to the cell cycle in endometrial cancer patients. Adv Med Sci.

[12] Chatterjee S, Gupta D, Caputo TA, Holcomb K (2016). Disparities in gynecological malignancies. Front Oncol.

[13] Hanna J, Hossain GS, Kocerha J (2019). The potential for microRNA therapeutics and clinical research. Front Genet.

[14] Delangle R, de Foucher T, Larsen AK (2019). The use of microRNAs in the management of endometrial cancer: a meta-analysis. Cancers.

[15] Xie W, Qin W, Kang Y, Zhou Z, Qin A (2016). MicroRNA-340-3p inhibits tumor cell proliferation and induces apoptosis in endometrial carcinoma cell line RL 95–2. Med Sci Monit: Int Med J Exp Clin Res.

[16] Raychaudhuri M, Bronger H, Buchner T, Kiechle M, Weichert W, Avril S (2017). MicroRNAs miR-7 and miR-340-3p predict response to neoadjuvant chemotherapy in breast cancer. Breast Cancer Res Treat.

[17] Mohammadi Yeganeh S, Vasei M, Tavakoli R, Kia V, Paryan M (2017). The effect of miR-340-3p over-expression on cell-cycle-related genes in triple-negative breast cancer cells. Eur J Cancer Care.

[18] Liang Y, Zhang P, Li S, Li H, Song S, Lu B (2018). MicroRNA-873-5p.2 acts as a tumor suppressor in esophageal cancer by inhibiting differentiated embryonic chondrocyte expressed gene 2. Biomed Pharmacotherapy—Biomed Pharmacotherapie.

[19] Gao R, Cai C, Gan J, Yang X, Shuang Z, Liu M, Li S, Tang H (2015). miR-1236-5p down-regulates alpha-fetoprotein, thus causing PTEN accumulation, which inhibits the PI3K/Akt pathway and malignant phenotype in hepatoma cells. Oncotarget.

[20] Zhang Q, Miao S, Li C, Cui K, Ge Q, Chen Z (2018). S-phase kinase-associated protein 2 impairs the inhibitory effects of miR-1236-3p on bladder tumors. Am J Transl Res.

[21] Hu B, Ying X, Wang J (2014). Identification of a tumor-suppressive human-specific microRNA within the FHIT tumor-suppressor gene. Can Res.

[22] Emmadi R, Canestrari E, Arbieva ZH, Mu W, Dai Y, Frasor J, Wiley E (2015). Correlative analysis of miRNA expression and oncotype Dx recurrence score in estrogen receptor positive breast carcinomas. PLoS ONE.

[23] Ghosh T, Varshney A, Kumar P, Kaur M, Kumar V, Shekhar R, Devi R, Priyanka P, Khan MM, Saxena S (2017). MicroRNA-874-3p-mediated inhibition of the major G1/S phase cyclin, CCNE1, is lost in osteosarcomas. The J Biol Chem.

[24] Wang L, Gao W, Hu F, Xu Z, Wang F (2014). MicroRNA-874-3p inhibits cell proliferation and induces apoptosis in human breast cancer by targeting CDK9. FEBS Lett.

